# Recent advances in machine learning methods for predicting LncRNA and disease associations

**DOI:** 10.3389/fcimb.2022.1071972

**Published:** 2022-11-30

**Authors:** Jianjun Tan, Xiaoyi Li, Lu Zhang, Zhaolan Du

**Affiliations:** Department of Biomedical Engineering, Faculty of Environment and Life, Beijing International Science and Technology Cooperation Base for Intelligent Physiological Measurement and Clinical Transformation, Beijing University of Technology, Beijing, China

**Keywords:** lncRNA, human diseases, lncRNA-disease associations, machine learning methods, predictive models

## Abstract

Long non-coding RNAs (lncRNAs) are involved in almost the entire cell life cycle through different mechanisms and play an important role in many key biological processes. Mutations and dysregulation of lncRNAs have been implicated in many complex human diseases. Therefore, identifying the relationship between lncRNAs and diseases not only contributes to biologists’ understanding of disease mechanisms, but also provides new ideas and solutions for disease diagnosis, treatment, prognosis and prevention. Since the existing experimental methods for predicting lncRNA-disease associations (LDAs) are expensive and time consuming, machine learning methods for predicting lncRNA-disease associations have become increasingly popular among researchers. In this review, we summarize some of the human diseases studied by LDAs prediction models, association and similarity features of LDAs prediction, performance evaluation methods of models and some advanced machine learning prediction models of LDAs. Finally, we discuss the potential limitations of machine learning-based methods for LDAs prediction and provide some ideas for designing new prediction models.

## 1 Introduction

Bioinformatics and transcriptomics analyses have shown that only a few genes in the human genome that encode proteins, and that more than 98% of human genes have no ability to encode proteins (Pertea, 2012). More and more studies have shown that non-coding RNAs (ncRNAs), in particular, long non-coding RNAs (lncRNAs) with a length of more than 200 nucleotides play an important role in physiological processes, such as epigenetic regulation, cell differentiation, cell cycle regulation and immune response at various stages of life (Chen et al., 2017). In addition, the disorders and mutations of lncRNAs are associated with many complex human diseases, such as neurological diseases (Johnson, 2012), cardiovascular diseases (Congrains et al., 2012), Alzheimer’s disease (Faghihi et al., 2008), leukemia (Calin et al., 2007) and various cancers (Pibouin et al., 2002; Su et al., 2015; Wu et al., 2022). Therefore, identifying the relationship between lncRNAs and diseases not only contributes to biologists’ understanding of disease mechanisms, but also provides new ideas and solutions for disease diagnosis, treatment, prognosis and prevention.

The identification of novel lncRNA-disease associations (LDAs) has attracted more and more attention and become an important topic in the field of medicine. At present, the association between a large number of lncRNAs and human diseases remains to be confirmed. Although biological experiments and clinical methods are effective and reliable for identifying LDAs, they are time-consuming, and expensive. In order to solve these problems, high quality computational methods have become the first choice for studying LDAs prediction, and machine learning models have achieved good results in LDAs prediction. There have been some reviews on LDAs prediction. For example, Long non-coding RNAs and complex diseases: from experimental results to computational models (Chen et al., 2017). Chen et al. review the function of lncRNAs, five important lncRNA-related diseases, five key disease-related lncRNAs, and some important lncRNA-related sequences, expressions, functions and other public databases, and they also introduce machine learning-based models, biological network-based models, and models that do not rely on known lncRNA-disease associations. These reviews of lncRNA-disease association prediction models are helpful for researchers in related fields to better understand the association between lncRNAs and diseases, and to design better models to improve prediction performance based on previous research basis and prospects for future research directions.

This review describes recent advances in machine learning methods for predicting lncRNA and disease associations. Firstly, some human diseases in LDAs prediction models are summarized to help understand the mode of action of lncRNAs in human diseases. Secondly, the association and similarity characteristics of LDAs prediction are summarized, which provide some methods for constructing input features for prediction models. Thirdly, the performance evaluation method of LDAs prediction model is briefly introduced. This paper focuses on some advanced machine learning-based LDAs prediction models in the past few years, these models are divided into two categories: conventional machine learning-Based models, deep learning-based models. The calculation process of some methods is described in detail, and the advantages and disadvantages of these methods are briefly explained. Finally, we discuss the potential limitations of machine learning-based methods for predicting LDAs and provide some ideas for designing new prediction models.

## 2 Associated diseases in prediction models

Mutations and dysregulation of lncRNAs can lead to the development of various complex human diseases, the identification of LncRNA and disease associations helps to understand the function of lncRNAs in diseases. In order to help understand the mode of action of lncRNAs in human diseases, some human diseases studied in LDAs prediction models are introduced as follows.

### 2.1 Osteosarcoma

Osteosarcoma is one of the most common primary bone malignant tumors originating, and it originates primarily the metaphysis of the long bones. The incidence of osteosarcoma is high in children and young people, which seriously threatens the health and life of children and adolescents (Yang et al., 2020). Although the clinical treatment of osteosarcomas such as chemotherapy and surgery has been improved, the prognosis of patients with osteosarcoma is still very poor, and the research on the molecular mechanism of osteosarcoma is still lacking (Hattinger et al., 2016). Therefore, it is urgent to study the pathogenesis of osteosarcoma and improve its clinical treatment effect. Studies have shown that many lncRNAs are involved in the formation and development of osteosarcoma. For example, LncRNA H19 inhibits the migration and invasion of human osteosarcoma cells by inhibiting the nuclear factor-KB pathway (Zhao and Ma, 2018). LncRNA PVT1 promotes osteosarcoma cell apoptosis and inhibits cell proliferation by regulating the expression of miR-195 (Zhou et al., 2016). GAS5 promotes the expression of aplasia Ras Homologue member I (ARHI) and inhibits the growth and Epithelial-Mesenchymal Transition of osteosarcoma cells by regulating the expression of miR-22 as a molecular sponger (Ye et al., 2017).

### 2.2 Lung cancer

Lung cancer is a kind of cancer, which has become the leading cause of cancer death worldwide in recent years (Wang et al., 2022c). In terms of histopathology, lung cancer is mainly divided into non-small cell lung cancer (NSCLC, about 80%) and small cell lung cancer (SCLC, about 20%) (White et al., 2014). Although chemotherapy and radiotherapy have certain therapeutic effects on lung cancer patients, in recent years, the 5-year survival rate of lung cancer after diagnosis is only about 15%, which is far lower than other types of cancer (Gutschner et al., 2013). Recent studies have shown that lncRNAs are involved in the key cellular processes of lung cancer, and are also related to the prognosis and advanced pathological staging of lung cancer patients (Loewen et al., 2014). LncRNA GAS5 inhibits the proliferation and metastasis of lung cancer cells through miR-205/PTEN axis (Dong et al., 2019). LncRNA XIST promotes the proliferation and migration of non-small cell lung cancer cells by regulating the expression of sponge miR-16 and CDK8 (Zhou et al., 2019). In addition, lncRNA UCA1 can regulate the proliferation and invasion of lung cancer cells and induce apoptosis, and UCA1 can be used as an important therapeutic target to inhibit lung cancer (Jun et al., 2018).

### 2.3 Gastric cancer

Gastric cancer is one of the most common malignant tumors in the world with high morbidity and mortality, which can be caused by factors such as diet, age and genetics (Smyth et al., 2020). There is more and more evidence that lncRNAs involvement in the mechanism of gastric cancer is of great significance for the early diagnosis, prognosis and treatment of gastric cancer. LncRNA HOTAIR can significantly inhibit the migration and invasion of gastric cancer cells (Xue et al., 2018). Overexpression of lncRNA BCYRN1 can directly up-regulate the expression of miR-204-5p and promote the development of gastric cancer (Zhai and Li, 2019). Moreover, the expression of HOTAIRM1 can inhibit the activity of GC cells by inhibiting the PI3K/AKT pathway in gastric cancer (Lu et al., 2019).

### 2.4 Prostate cancer

Prostate cancer is a common epithelial malignant tumor of the prostate in the urinary and reproductive systems. It is the most common malignant tumor in the male population and the second leading cause of cancer-related death in men (Saini, 2016). Studies have shown that lncRNAs associated with prostate cancer may contribute to the prevention and treatment of prostate cancer (Cui et al., 2020). LncRNA TTTY15 is upregulated in most prostate cancer tissues and can promote the development of prostate cancer through sponge let-7 (Xiao et al., 2019). In addition, different variants of CDKN2B-AS1 are associated with prostate cancer, and CCAT2 expression is upregulated in prostate cancer patients and affects prostate cancer development by altering the epithelial-mesenchymal transition (Fehringer et al., 2016; Zheng et al., 2016).

### 2.5 Breast cancer

Breast cancer is the most common cancer in women worldwide and the second leading cause of cancer death in women (Donahue and Genetos, 2013; Shi et al., 2022a). The traditional diagnosis of breast cancer is based on the shape, size, and nature of the breast mass as well as regional lymph node mass and other features. Accumulating studies have shown that lncRNAs such as MALAT1 and ZFAS1 are closely related to breast cancer. For example, MALAT1 leads to the epithelial-to-mesenchymal transition program through a phosphatidylinositide-3 kinase-AKT pathway in breast cancer, and thus MALAT1 is significantly downregulated in breast cancer tissues and cell lines (Zhao et al., 2014). In addition, ZFAS1 overexpression can significantly inhibit cell proliferation by causing cell cycle arrest and inducing apoptosis in breast cancer cells (Fan et al., 2018).

### 2.6 Cervical cancer

Early symptoms of cervical cancer are difficult to be detected, and it is one of the gynecological tumors with the highest cancer-related mortality worldwide (Adey et al., 2013). It is very important to study the complex pathogenesis of cervical cancer and diagnose its prognostic biomarkers. Many lncRNAs have been proven to be novel regulators in various biological processes, playing a crucial role in the occurrence and progression of cervical cancer and other cancers (Peng et al., 2016). For example, UCA1 up-regulates and inhibits the growth of cervical cancer cells in cervical cancer, which is a potential target for the treatment of cervical cancer cells (Yan et al., 2018). In addition, serum PVT1 can accurately distinguish cervical cancer patients from healthy controls (Yang et al., 2016).

### 2.7 Hepatocellular carcinoma

Hepatocellular carcinoma (HCC) is a malignant tumor of liver parenchymal cells with a poor prognosis. Since many HCC patients are already in the advanced stage of cancer at the time of diagnosis, it is urgent to understand the principle of HCC and improve the ability for early diagnosis (Men et al., 2020; Shi et al., 2022b). Studies have shown that lncRNAs have an important impact on human HCC. LncRNA TP73-AS1 is upregulated in HCC tissues and cell lines, competing with HMGB1 for Mir-200A binding to inhibit its expression, and subsequently upregulating HMGB1/RAGE expression to promote HCC cell proliferation (Li et al., 2017). The up-regulation of lncRNA-SOx2-OT promotes the metastasis of hepatocellular carcinoma, and the high expression of lncrNA-SOX2-OT is related to histological grade, TNM stage and venous invasion (Shi and Teng, 2015).

### 2.8 Glioma

Glioma is one of the most common brains and central nervous system tumors, accounting for about 80% of malignant brain tumors, characterized by aggressive vascularization (Khasraw et al., 2014). Despite the continuous improvement of various treatment methods such as surgery, radiotherapy and chemotherapy, the overall survival time of glioma patients is only about 12-14 months after diagnosis (Wang et al., 2015). Recent studies have shown that lncRNAs play an important role in the pathogenesis of glioma (Bian et al., 2015). The expression level of lncRNA MALAT1 is significantly correlated with the overall survival of glioma patients, which can be used as a persuasive prognostic biomarker for glioma patients (Ma et al., 2015). XIST expression was significantly up-regulated in glioma tissues, and negatively correlated with Mir-137 expression. This result revealed a new XIST-Mir-137-RAC1 pathway regulatory axis in the pathogenesis of glioma (Wang et al., 2017). In addition, Gas5 increased the expression of glioma inhibitor Bcl-2 modifier and Plexin C1 by directly targeting and reducing the expression of Mir-222 (Zhao et al., 2015).

## 3 Association and similarity characteristics

**Figure 1:**
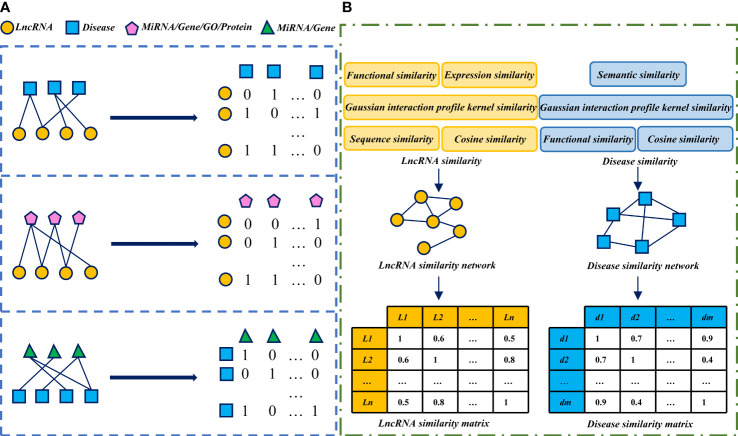
The process of network and matrix construction of association and similarity: **(A)** Construct association network and matrix based on known association data obtained from the database. In the construction of the association matrix. If a data pair is associated, the matrix element sets to 1, otherwise, 0. **(B)** The similarity network and matrix of LncRNA/disease were constructed by calculating the similarity score of LncRNA/disease.

Association network/matrix and similarity network/matrix are commonly used as input features in lncRNA-disease association prediction models. Some models integrate multiple association data, lncRNA and disease similarity features. These association data and similarity features complement each other, and more lncRNA/disease characteristic information can be obtained. It will greatly improve the predictive performance of the model. The association and similarity network and matrix construction process used in the lncRNA-disease association prediction model is shown in **Figure 1**.

### 3.1 Association characteristics

In addition to lncRNA-disease association data used in the lncRNA-disease association prediction model, lncRNA-miRNA association, lncRNA-gene association, lncRNA-Gene Ontology (Gene Ontology, GO) association and lncRNA-protein association are also the original association characteristic data of lncRNAs. The original association characteristic data of diseases include disease-miRNA association and disease-gene association, etc (Li et al., 2021). The lncRNA-disease associations can be downloaded from LncRNADisease (Bao et al., 2019) and Lnc2Cancer (Gao et al., 2021). The known association of lncRNA with miRNA, Gene, GO and protein can be downloaded from StarBase v2.0 (Li et al., 2014), LncRNA2Target (Cheng et al., 2019), GeneRIF (Lu et al., 2007) and NPInter v4.0 (Teng et al., 2020), respectively. Disease associations with miRNAs and Gene can be downloaded from HMDD (Huang et al., 2019) and DisGeNET (Piñero et al., 2017), respectively. After downloading the known association data from databases, the corresponding association networks and matrices can be constructed as the original features of the lncRNA-disease association prediction model.

### 3.2 Similarity characteristics

Since lncRNAs with similar functions tend to exhibit similar associations with diseases, calculating the similarity between lncRNAs will help identify potential associations between diseases and lncRNAs. Similar diseases exhibit similar interaction patterns with lncRNAs, and the similarity between diseases also provides important information for predicting potential lncRNA-disease associations. Common lncRNA similarities include lncRNA functional similarity, lncRNA expression similarity, lncRNA sequence similarity, lncRNA Gaussian interaction profile kernel similarity and lncRNA cosine similarity (van Laarhoven et al., 2011; Chen and Yan, 2013; Chen, 2015; Xie et al., 2019; Yang and Li, 2021). Commonly used disease similarity includes disease Gaussian interaction profile kernel similarity, disease cosine similarity, disease semantic similarity and disease functional similarity (Schlicker et al., 2010; Wang et al., 2010; van Laarhoven et al., 2011; Xie et al., 2019). These similarity calculation methods can also be used by other studies, such as microbe-associated diseases prediction (Yin et al., 2022) and miRNA-disease association prediction (Chen et al., 2018b). The similarity calculation methods in the lncRNA-disease association prediction model are summarized as follows.

#### 3.2.1 LncRNA function similarity

Previous studies established the LFSCM model (Chen, 2015) based on the hypothesis that functionally similar lncRNAs tend to interact with similar miRNAs, and similar miRNAs tend to be associated with similar diseases. Disease semantic similarity between diseases is calculated according to the direct acyclic graphs (DAGs) of diseases. The disease data sets associated with each miRNA were determined and the similarity between them was calculated as miRNA functional similarity. The functional similarity of lncRNA was calculated according to the interaction between miRNA functional similarity and lncRNA functional similarity. The lncRNA functional similarity matrix is FS, where the element *FS*(*i, j*) in row *i* and column *j* is the functional similarity between lncRNA *l*(*i*) and *l*(*j*) obtained by the LFSCM similarity calculation model.

#### 3.2.2 LncRNA expression similarity

The lncRNA expression profiles generated by RNA-seq technology can be downloaded from ArrayExpress (Parkinson et al., 2007). The expression similarity between two lncRNAs can be obtained by calculating the Spearman correlation coefficient between expression profiles (Chen and Yan, 2013). The expression similarity matrix of lncRNA is assumed to be ES, where the element *ES*(*i, j*) in row *i* and column *j* is the expression similarity between lncRNA *l*(*i*) and *l*(*j*) ranging from 0 to 1.

#### 3.2.3 LncRNA sequence similarity

LncRNA sequence data can be downloaded from LncRNADisease, and the Needleman-Wunsch alignment method (NW) (Needleman and Wunsch, 1970) is used to calculate the sequence similarity of lncRNA. SS is defined as the sequence similarity matrix of lncRNA, then the sequence similarity between lncRNA *l*(*i*) and *l*(*j*) is *SS*(*i, j*). *SS*(*i, j*) is the element in row i and column j of SS.

#### 3.2.4 Disease semantic similarity

Disease semantic similarity was calculated using disease classification data in MeSH database (Wang et al., 2010). Each disease is numbered according to its tree structure in the MeSH database to form a directed acyclic graph (DAG). Each disease can be transformed into a homologous directed acyclic graph (DAG). For example, the DAG of disease d can be expressed as *DAG*(*d*) = (Anc(d), E(d)),and Anc(d), and represents the node set including nodes and their ancestors. *E*(*d*) is the edge directly connected between the parent node and the child node, it shows the correlation between different diseases. According to the DAG graph of disease, the semantic value contribution of disease *d* to other diseases and the semantic value of disease *d* itself is calculated. The more ancestral diseases the two diseases share in their DAG, the higher the semantic similarity value they obtain.

#### 3.2.5 Disease functional similarity

A computational model based on the directed acyclic graph (DAG) was proposed to measure the semantic similarity of GO (Wang et al., 2007). According to a large-scale functional association network of human genes provided by predecessors (Lee et al., 2011), it uses the correlation log-likelihood score (LLS) for each edge to measure the strength of association between any two genes. According to the log-likelihood score of genes, the gene similarity network was established by simple processing. In order to obtain a more accurate functional similarity network of diseases, Jaccard similarity coefficient was used to calculate the functional similarity of diseases from the perspectives of disease-GO association and disease-gene association (Xiao et al., 2018).

#### 3.2.6 Gaussian interaction profile kernel similarity for LncRNA and disease

Gaussian interaction profile kernel similarity is a common feature of lncRNA and disease. The similarity of Gaussian interaction profile kernel of disease was calculated as follows: Firstly, an adjacency matrix was constructed using the association data between lncRNA and disease. The columns of the matrix represent lncRNAs and the rows represent diseases. Then, the Radial Basis Function (RBF) Gaussian kernel function was applied to the adjacency matrix to obtain the similarity matrix of the Gaussian interaction profile kernel of the disease (van Laarhoven et al., 2011; Chen et al., 2018a). The kernel similarity of Gaussian interaction profile of lncRNA was calculated in the same way as that of disease. An adjacency matrix was constructed using the association data between lncRNA and disease. The columns of the matrix represent diseases and the rows represent lncRNAs. Then, the Radial Basis Function (RBF) Gaussian kernel function was applied to the adjacency matrix to obtain the similarity matrix of the Gaussian interaction profile kernel of lncRNA.

#### 3.2.7 Cosine similarity for lncRNA and disease

According to previous studies, the KMDR calculation model, proposed the cosine similarity measure in the collaborative filtering recommendation algorithm (Adomavicius et al., 2005), which was successfully applied to miRNA-disease association prediction (Li et al., 2018). Inspired by the above algorithm, SKF-LDA calculation model (Xie et al., 2019) proposed and successfully applied cosine similarity to lncRNA-disease association prediction. The basic assumption of lncRNA cosine similarity is that if lncRNAs *l_i_* and *l_j_* are similar to each other, then *A*(*i,:*) and *A*(*j,:*) in lncRNA-disease association matrix A are similar to each other. Where, *A*(*i,:*) is the row *i* of lncRNA-disease association matrix A, which contains the relationship between all diseases and lncRNA *l_i,_A*(*j,:*) is the row *j* of lncRNA-disease association matrix A, which contains the relationship between all diseases and lncRNA *l_i_*. Cos (*A*(*i*,:)) represents the cosine similarity score between lncRNA *l_i_* and lncRNA *l_j_*. The above lncRNA hypothesis is also applicable to diseases. If disease *d_i_* and disease *d_j_* are similar to each other, then *A*(: *i*) and *A*(: *j*) in lncRNA-disease association matrix A are similar to each other. *A*(: *i*) represents the column *i* of lncRNA-disease association matrix A, which contains the relationship between all lncRNAs and disease *d_i_*. *A*(: *j*) represents the column *j* of lncRNA-disease association matrix A, which contains the relationship between all lncRNAs and disease *d_j_*. Cos [*A*(: *i*), *A*(:*j*)] represents the cosine similarity score between disease *d_i_* and *d_j_*.

## 4 Performance evaluation

The predictive performance of lncRNA-disease association prediction models is usually evaluated by K-fold cross validation (K-CV) or leave-one-out cross validation (LOOCV). K-CV means that the original sample data is divided into K groups on average, and each subset data is used as a validation set, and the remaining subset data of K-1 group is used as the training set to obtain K models. The average classification accuracy of the final validation set of these K models is used as the performance index of the classifier under this K-CV. 5-CV and 10-CV are the most commonly used. LOOCV takes one of the original N sample data as the test set, and the remaining N-1 sample data as the training set to obtain N models. Finally, the average classification accuracy of the final validation set of these N models is taken as the performance index of the classifier under LOOCV. LOOCV makes full use of all the sample information through N times prediction, and the result is the closest to the expected value of training the whole test set. Because of its high computational cost, it is not suitable for large sample data.

The evaluation indexes of lncRNA-disease association prediction model mostly used the receiver operating characteristic (ROC) curve, the area under ROC curve (AUC), Precision-Recall (PR) curve and the area under PR curve (AUPR). Sensitivity and specificity are two key indexes used in ROC curve. For the prediction of lncRNA-disease associations, sensitivity represents the proportion of a test that is correctly identified, while specificity represents the proportion of a test that is incorrectly considered to have an association. In this way, ROC curves are drawn using a true positive rate (sensitivity) and a false positive rate (1-specificity) by continuously changing the threshold. The area under the ROC curve is also commonly used to test performance. In general, AUC = 0.5 means the effect is random, and AUC = 1 means the effect is perfect. Precision and recall are two key indexes used in PR curve. For the prediction of lncRNA-disease association, the accuracy rate represents how many of the samples with a positive prediction are truly positive samples, while the recall rate represents how many of the positive examples in the sample are predicted correctly. PR curves are drawn using precision and recall by traversing different thresholds for comparison, and the area under the PR curve is also commonly used to test performance. Generally, the larger the area under the PR curve, the better the model performance.

## 5 Machine learning-based models

Several researchers have built a number of machine learning-based models to predict LDAs. The model based on machine learning trains the classifier according to the characteristics of the training samples to get the classifier with the function of predicting LDAs. These machine learning models have achieved good results in predicting LDAs. Predicting LDAs based on machine learning has attracted more and more researchers’ attention. This section describes some of the advanced machine learning-based LDAs prediction models, detailing the computational process of some methods. In this section, these machine learning-based LDAs prediction models are divided into two categories: conventional machine learning-based models, deep learning-based models.

### 5.1 Conventional machine learning-based models

Traditional machine-learning methods commonly used in LDAs prediction mainly include support vector machine (SVM), random forest (RF), extreme gradient boosting (XGBoost), Adaptive boosting (Adaboost), K-Nearest Neighbors(K-NN), Singular value decomposition (SVD), collaborative filtering (CF) algorithm, Laplacian Regularized Least Squares algorithm and some traditional matrix factorization and completion algorithms, etc.

**Figure 2:**
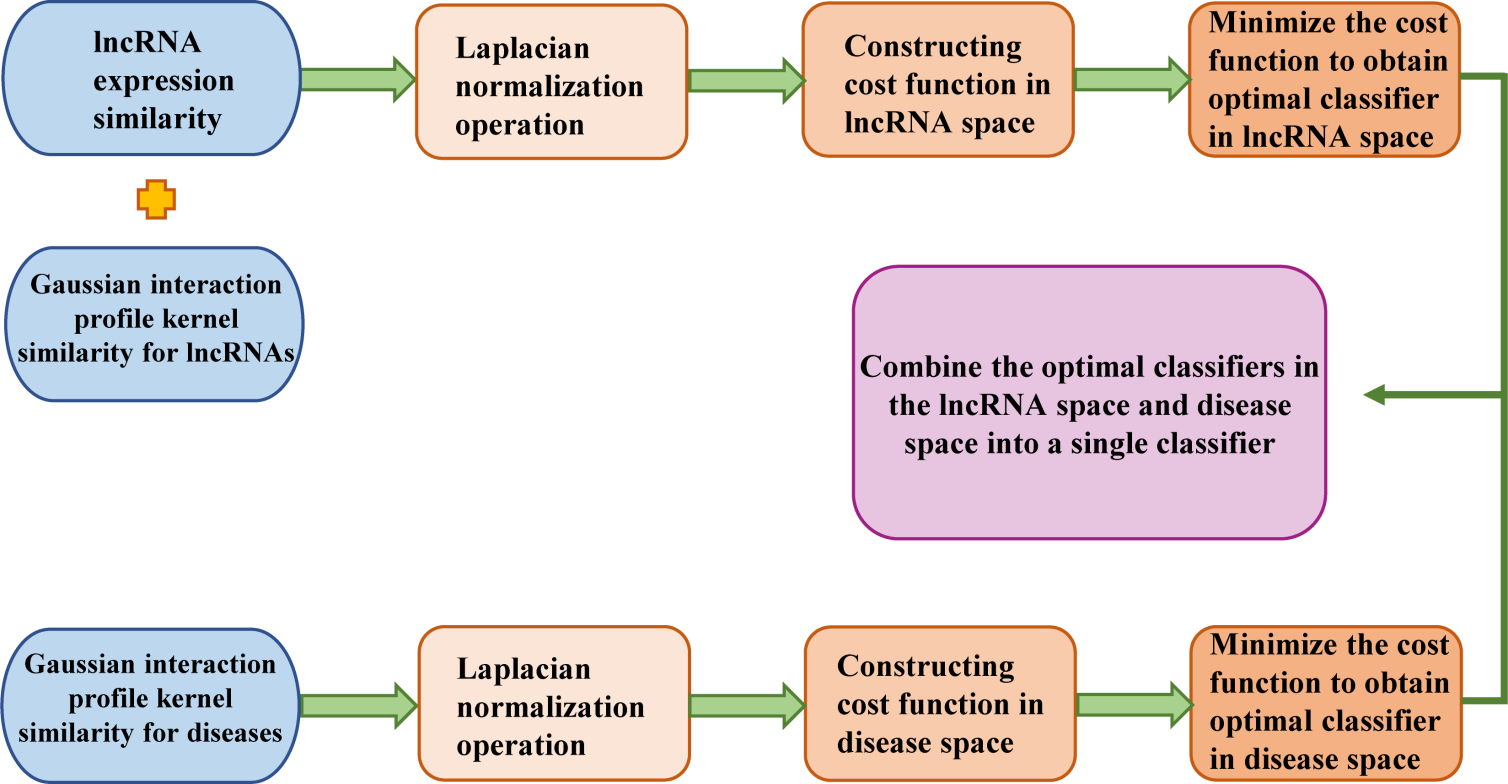
The flowchart of LRLSLDA is shown here, including the basic steps to predict potential disease-related lncRNAs based on LRLSLDA.

LRLSLDA (Chen and Yan, 2013) is the first lncRNA-disease association prediction model. This model is a semisupervised learning method developed in the Laplacian Regularized Least Squares framework. This method integrates known lncRNA-disease associations and lncRNA expression profiles to identify potentially disease-related lncRNAs. The process of predicting potential disease-associated lncRNAs based on LRLSLDA is shown in **Figure 2**. It does not require negative samples and can prioritize lncRNA-disease pairs for all diseases simultaneously. This method obtained an AUC of 0.7760 under leave-one-out cross validation, and laid the solid theoretical foundation for the study of lncRNA-disease association prediction. The code of LRLSLDA is freely available at: http://asdcd.amss.ac.cn/Software/Details/2.

Fu et al. propose a Matrix Factorization based LncRNA–Disease Association prediction model (MFLDA) (Fu et al., 2018). MFLDA decomposes data matrices of heterogeneous data sources into low-rank matrices *via* matrix tri-factorization, then select and integrate the data sources by assigning different weights to them. An iterative solution is further introduced to simultaneously optimize the weights and low-rank matrices. Finally, MFLDA uses the optimized low-rank matrices to reconstruct the lncRNA–disease association matrix and thus to identify potential associations. MFLDA achieves an area under the receiver operating characteristic curve (AUC) of 0.7408. MFLDA can also be adopted to predict associations between other biological entities. The source code for MFLDA is available at: http://mlda.swu.edu.cn/codes.php?name¼ MFLDA.

WGRCMF model introduces graph regularization constraints on the basis of collaborative matrix factorization to fully integrate the internal geometric structure of the data, and introduces a weight matrix to prevent unknown associations from affecting the final prediction matrix (Liu et al., 2021). This model can effectively predict potential lncRNA-disease associations by integrating known lncRNA-disease associations, lncRNA similarity matrix and disease similarity matrix. This model achieved an AUC value of 0.8556 by performing 30 times 10-fold cross-validation.

**Figure 3:**
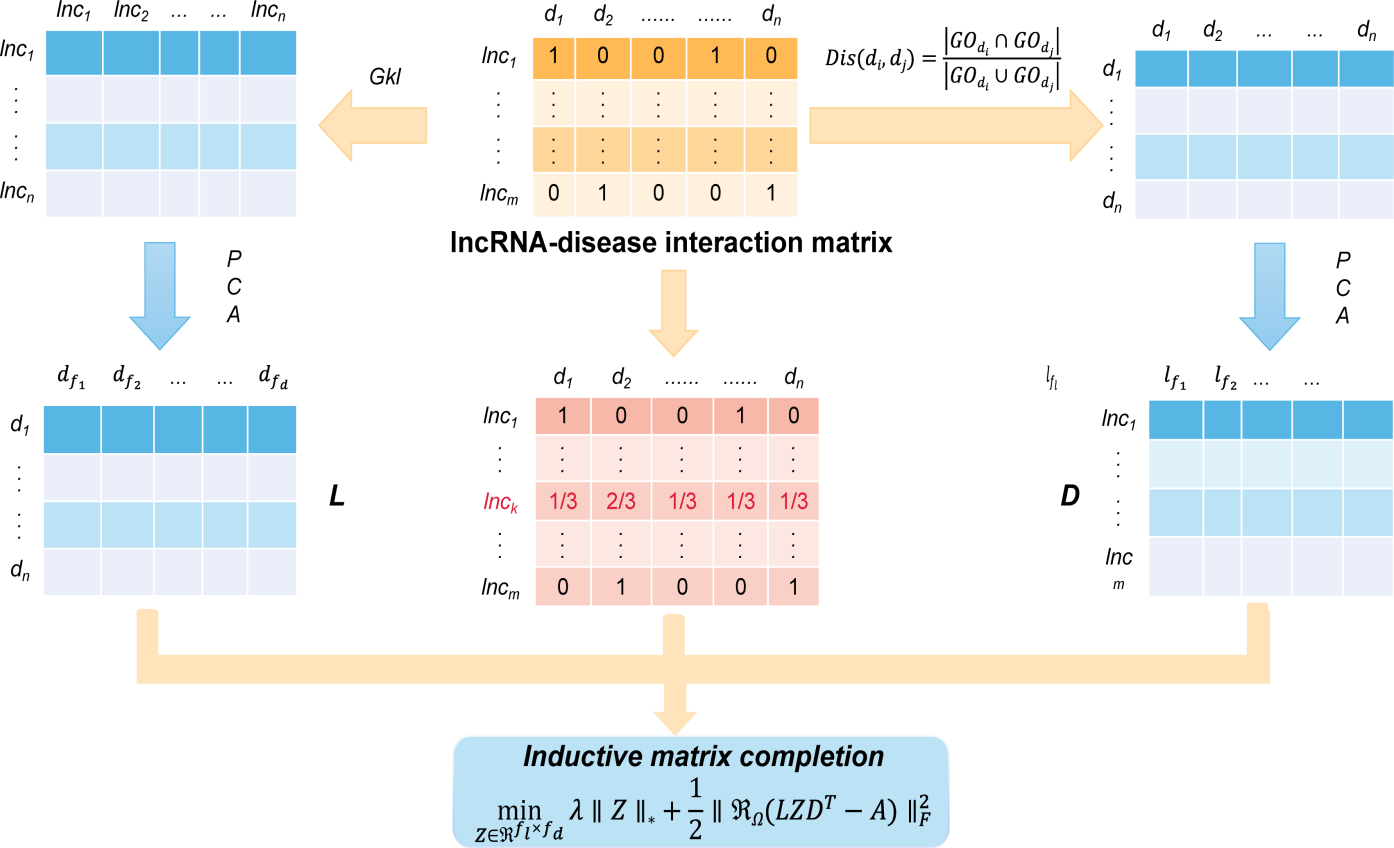
The workflow indicates that SIMCLDA predicts potential lncRNA–disease associations based on inductive matrix completion.

Lu et al. proposed SIMCLDA as an inductive matrix completion based to predict lncRNA disease interactions (Lu et al., 2018). Specifically, the method can be completed in five steps as shown in **Figure 3**. The first step of this method, based on the hypothesis that functionally similar lncRNA have similar patterns of interaction with the disease, using *Gkl*∈*R* ^*m*×*m*^ to define the potential feature space of lncRNA containing the feature matrix. Then, disease similarity was calculated using the method called Jaccard. Step 3, using singular value decomposition (SVD) to perform PCA to extract the primary feature vectors from *Gkl*∈*R* ^*m*×*m*^ and *Dis*∈*R* ^*n*×*n*^ , respectively. Step 4, based on the hypothesis that similar lncRNA interact with similar diseases, the interaction distribution of the new lncRNA was calculated using the mean of its neighbors’ interaction profiles. With the calculated interaction profiles, we were able to combine the previous interaction patterns of such new lncRNA neighbors and extract effective feature vectors. Finally, SIMCLDA uses the primary feature vector to complete the association matrix with inductive matrix completion and construct the interaction profile:


(1) $$\underset{Z\in {ℜ}^{f_{l}\times f_{d}}}{\min}\lambda ∥Z{∥}_{*}+\frac{1}{2}∥{ℜ}_{\text{Ω}}\left(LZD^{T}-A\right){∥}_{F}^{2}$$

where ∥*Z*∥_∗_ is the objective matrix to complete A which is defined as the sum of the singular values. the column vectors in A lie in the subspace spanned by the column vectors in L, and the row vectors in A lie in the subspace spanned by the column vectors in D. The computational results showed that SIMCLDA can effectively predict lncRNA-disease correlations with higher precision than previous methods. The results show that SIMCLDA achieves an AUC of 0.8526. In addition, case studies have shown that SIMCLDA can effectively predict candidate lncRNA for kidney cancer, gastric cancer and prostate cancer. The source code for SIMCLDA is available at: https://github.com//bioinfomaticsCSU/SIMCLDA.

**Figure 4:**
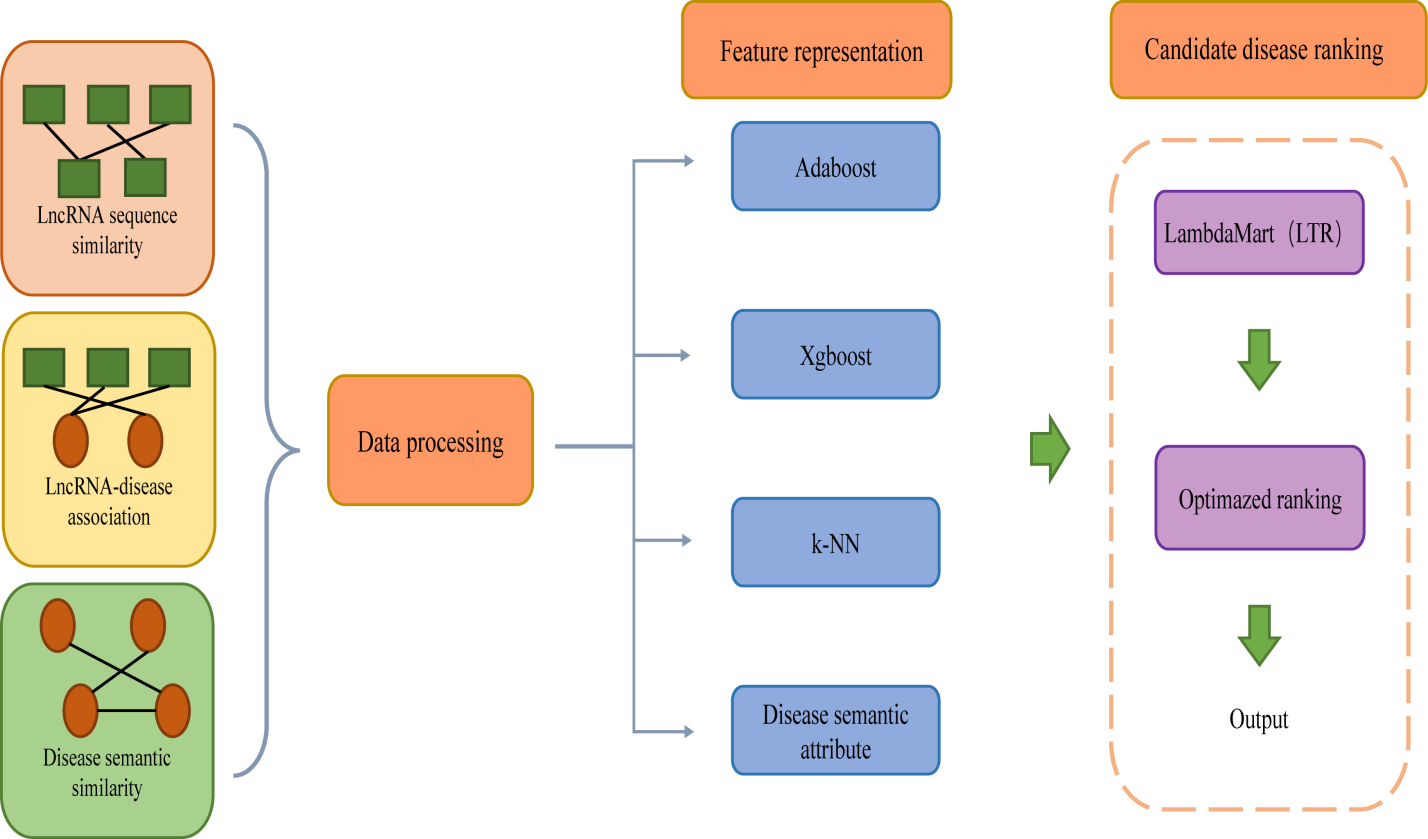
From the workflow, ilncDA-LTR integrating multi-source information into LTR to predict lncRNA-disease associations.

Wu et al. (Wu et al., 2022). introduced an integration model called iLncDA-LTR for predicting diseases related to newly detected lncRNAs (**Figure 4**). iLncDA-LTR is a model that integrates multi-source information into LTR to predict lncRNA-disease associations, including three steps: data processing, feature representation and candidate disease ranking. The first step of this model is data processing, disease semantic similarity, lncRNA sequence similarity and lncRNA-disease association matrix are collected, the semantic similarity between disease *d_i_* and disease *d_j_* is defined as *DSIM*(*di, dj*), the sequence similarity between lncRNA *l_i_* and lncRNA *l_i_* is defined as LSIM(*li, lj*), and similarly, lncRNA-disease association matrix is defined as *YER* ^nxm^, where n denotes the number of lncRNAs and m denotes the number of diseases. In the second step of this model, Adaptive boosting (Adaboost), Extreme gradient boosting (Xgboost) and k-nearest neighbor (k-NN) are selected as component methods. Afterward, the features of lncRNA-disease pairs are constructed, which can be formulated. Then integrate the disease semantic attribute features of *DSIM*(*dj*, :) into *F^LTR^* (*li*, *dj*), where *F^LTR^* (*li*, *dj*) is the feature vector of pair between lncRNA *l_i_* and disease *d_j_*. Compared with the features of disease semantic attributes, features obtained by various compositional methods integrate more evidence. Finally, LambdaMart algorithm belonging to the listwise approach of LTR is used to train the LTR model, and Normalized Discounted Cumulative Gain (NDCG) and Mean Average Precision (MAP) are integrated into the loss function of LambdaMart to improve the ranking quality. For querying lncRNAs, fed features into the trained LTR model, the returned ranked list of diseases is the prediction results. The method integrates various biological information into the framework of LTR for identifying diseases associated with newly detected lncRNAs. By comparing iLncDA-LTR with other methods, iLncDA-LTR achieves an AUC of 0.951, which is higher than previous prediction methods, and obtains the best performance. In the future, LTRs may also be used to predict unknown disease-drug associations among other similar problems. The web server of iLncDA-LTR is at: http://bliulab.net/iLncDA-LTR/.

**Figure 5:**
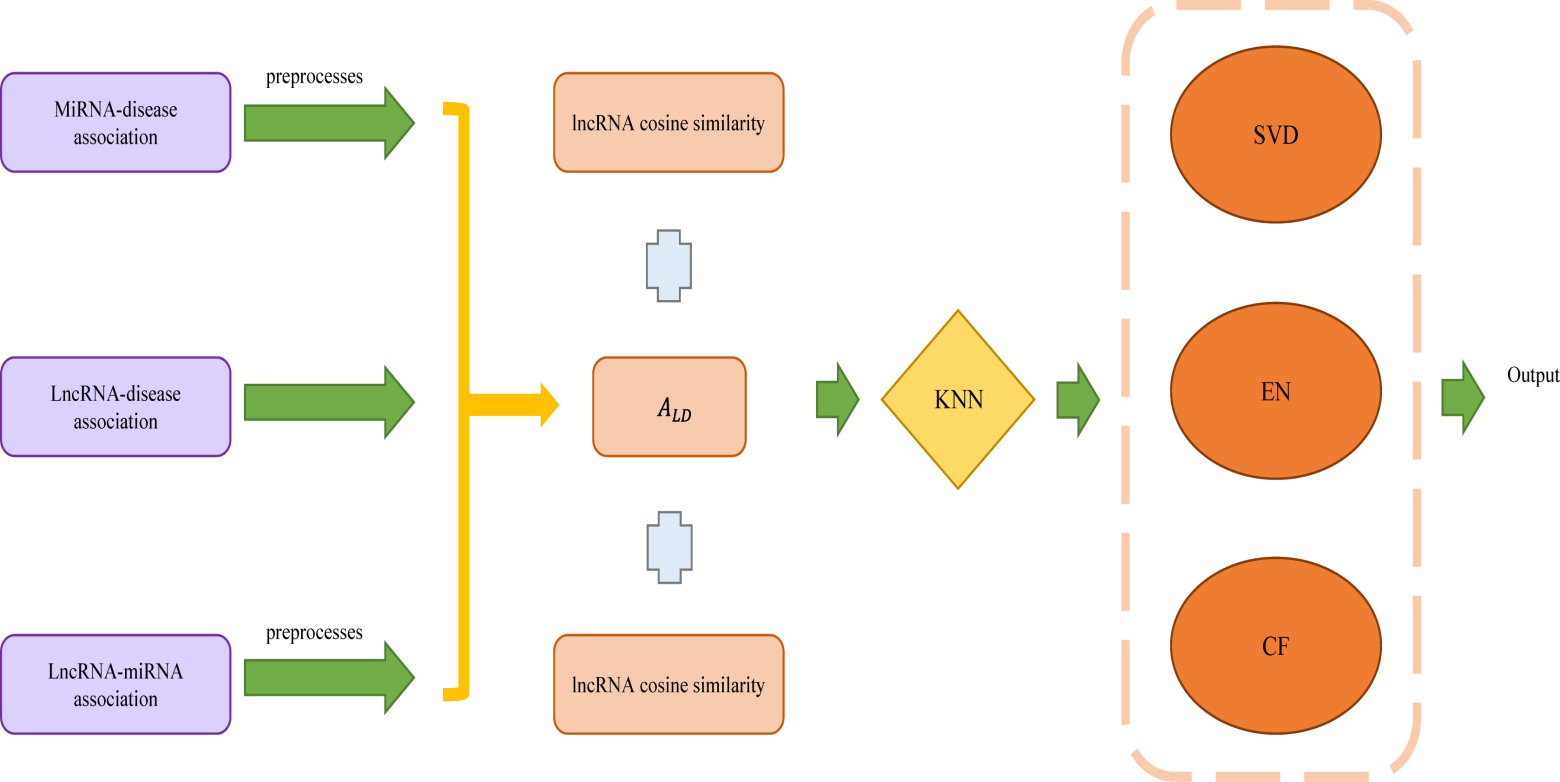
The flowchart of ENCFLDA to predict the association of lncRNAs with diseases based on elastic network, matrix decomposition and collaborative filtering.

Wang et al. established a lncRNA−disease association prediction model (ENCFLDA) combining elastic network, matrix decomposition and collaborative filtering to predict the association of unknown lncRNAs with diseases (**Figure 5**) (Wang et al., 2022a). This method obtains the existing miRNA-disease association datasets with lncRNA-disease association datasets and lncRNA-miRNA association datasets, then preprocesses the data to construct the miRNA-disease adjacency matrix AMD and the lncRNA-miRNA adjacency matrix ALM. Using AMD and ALM to calculate the lncRNA-disease association matrix ALD. Afterward, the calculated disease cosine similarity and lncRNA cosine similarity are combined with ALD to make the matrix less sparse using the weighted KNN. This is followed by the construction of the ENCFLDA model: Firstly, decomposes ALD into two different matrices using matrix decomposition, and then updates the matrix by combining it with an elastic net algorithm. Finally, the score matrix is calculated by the random gradient descent method and the lncRNA-based collaborative filtering algorithm. In addition, by using Leaving One Cross Validation (LOOCV) to compare ENCFLDA with the current more advanced mode, ENCFLDA achieved the highest AUC value of 0.9148. The results indicate that ENCFLDA model outperformed the other models in terms of prediction. The case study also verified the accuracy of the model. ENCFLDA model not only removes invalid features, but also has good stability, and has better results for sparse models with few weights. The source code for ENCFLDA is available at: https://github.com/arejay1998/ENCFLDA.

### 5.2 Deep learning-based models

Deep learning-based methods used in LDAs prediction mainly include CNN, recurrent neural network (RNN), graph embedding (GAE), Stacked Denoising Auto Encoder (SDAE), Graph neural network (GNN) and graph convolution network (GCN), etc.

Wu et al. proposed a machine learning-based classification method (GAERF) to identify disease-associated lncRNAs by graph embedding (GAE) and random forest (RF) (Wu et al., 2021). Superior performance is achieved by GAERF. Firstly, the LMD network is constructed based on the association, interaction and similarity of integrated lncRNAs, diseases and miRNAs, which can make full use of various data to characterize potential LDAs. Secondly, the deep feature representation of nodes in the network is extracted using graph embedding method, which preserves the topology of nodes and internal information of nodes in the network. GAERF achieves superior performance due to the ensemble learning method.

VADLP is used to extract, encode, and adaptively integrate a predictive model for multi-layer representation (Sheng et al., 2021). A three-level heterogeneous graph with inter-layer and intra-layer edges weighting is constructed to facilitate node attribute embedding and pairwise topology extraction for random wandering, and the model defines three representations, including node attributes, pairwise topology, and feature distributions. And an attentional representation-level integration module is constructed to adaptively fuse these three representations for lncRNA-disease association prediction. The model has advantages in discovering true lncRNA-disease associations and returning them as top-ranked candidates.

Zhou et al. propose a novel lncRNA-disease association prediction method LDAformer based on topological feature extraction and Transformer encoder (Zhou et al., 2022). This method constructs the heterogeneous network by integrating the associations between lncRNAs, diseases and micro RNAs (miRNAs). After the similarity calculation, inter-class associations and intra-class similarities are concatenated into the lncRNA-disease-miRNA weighted adjacency matrix. Then design a topological feature extraction process to capture multi-hop pathway information. Finally adopt a predictor based on the self-attention encoder to learn the interdependencies between pathways globally. LDAformer can accurately discover potential lncRNA–disease pairs in practical cases. The codes of LDAformer are available at https://github.com/EchoChou990919/LDAformer.

GCRFLDA is a prediction method based on graph convolution matrix complementation (Fan et al., 2022). The method constructs a graph using lncRNA-disease association information. Then, an encoder consisting of a conditional random field and an attention mechanism and a decoder layer are constructed to learn the effective embedding of nodes and score the lncRNA-disease association. Experimental results show that because the GCRFLDA model uses the LNF (Fan et al., 2020) method to fuse similarity information as edge information of nodes and incorporates the attention mechanism, the model has good potential relevance prediction and strong robustness. The model achieved high AUC in four benchmark datasets. In a case study of four diseases, the model found that 70 out of 80 predicted associated lncRNAs were confirmed in the literature. The code of GCRFLDA is available at https://github.com/jademyC1221/GCRFLDA.

**Figure 6:**
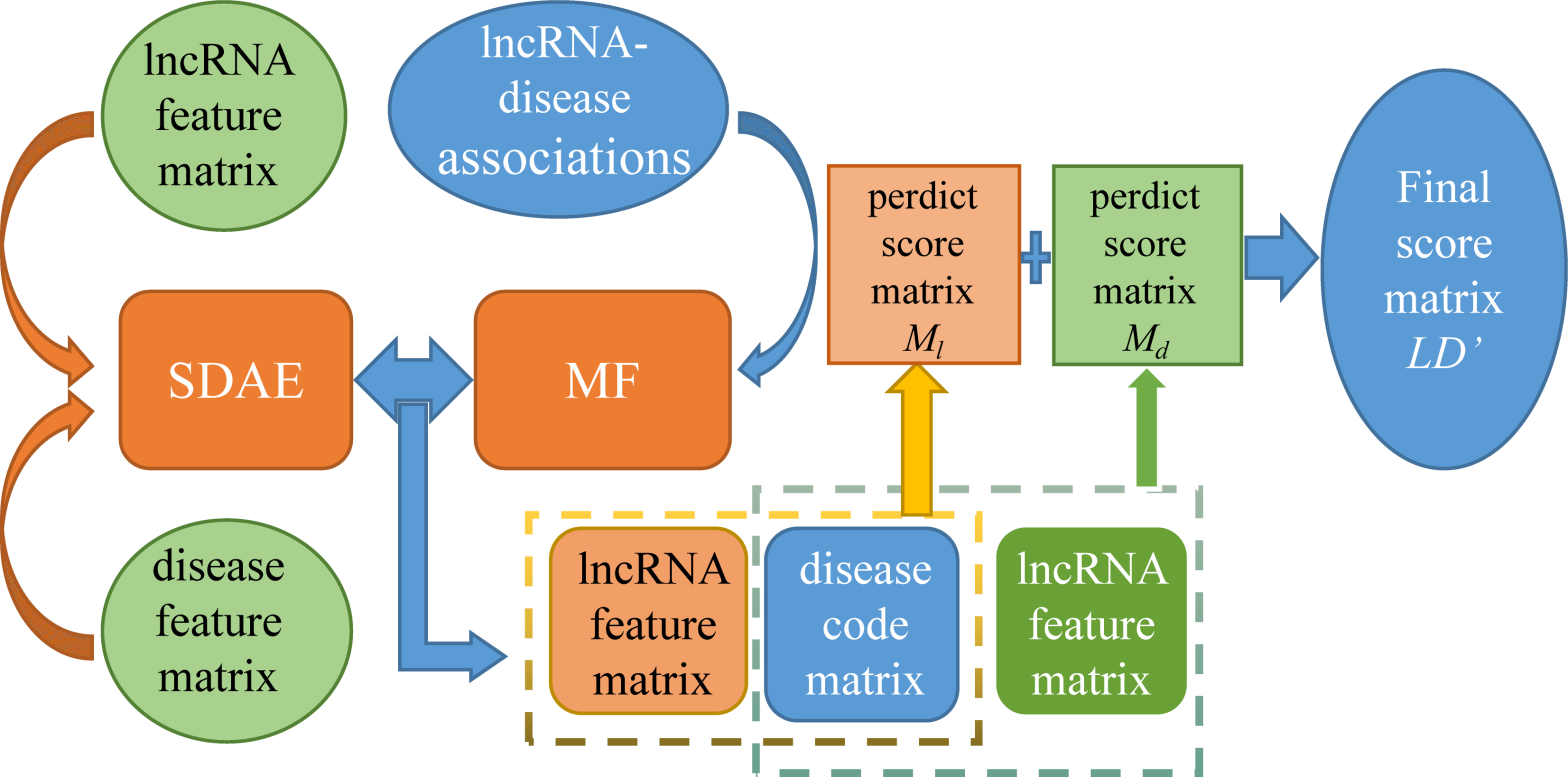
The flowchart of LDICDL for lncRNA-disease associations based on collaborative deep learning.

Lan et al. came up with a new method (**Figure 6**) called LDICDL to identify lncRNA-disease associations (Lan et al., 2022). LDICDL uses the stacked denoising autoencoder (SDAE), which is a feedforward neural network widely used in recommender systems to select lncRNA and disease features and reduce these features to k dimensions. Meanwhile, the method applies matrix factorization to predict lncRNA-disease association. The specific methods are as follows: firstly, we need to construct a hybrid model combining matrix decomposition and stored denoising autoencoder, input lncRNA features and disease features respectively. Then, we need to input *X_input l_*, X*_input d_* into the layer for encoding to get *X_encode l_* and *X_encode d_*, and finally enter the output layer to get *X_out l_* and *X_out d_* which is the encoding matrix of lncRNA and disease. According to the lncRNA feature matrix, the disease coding matrix, the disease feature matrix and the lncRNA coding matrix, respectively to predict the lncRNA-disease association scores. Finally, the final score of the disease association was calculated by averaging the scores. Based on collaborative deep learning, LDICDL overcomes the limitations of the substrate decomposition algorithm and builds hybrid models to predict the association of new lncRNAs and diseases. LDICDL evaluated the performance using ten-fold cross validation and obtained the AUC of 0.9134. To demonstrate the ability of LDICDL in identifying potential lncRNA-disease associations, a case study on osteosarcoma was performed with good results. The results show that LDICDL outperforms than other state-of-the-art methods in prediction performance.

**Figure 7:**
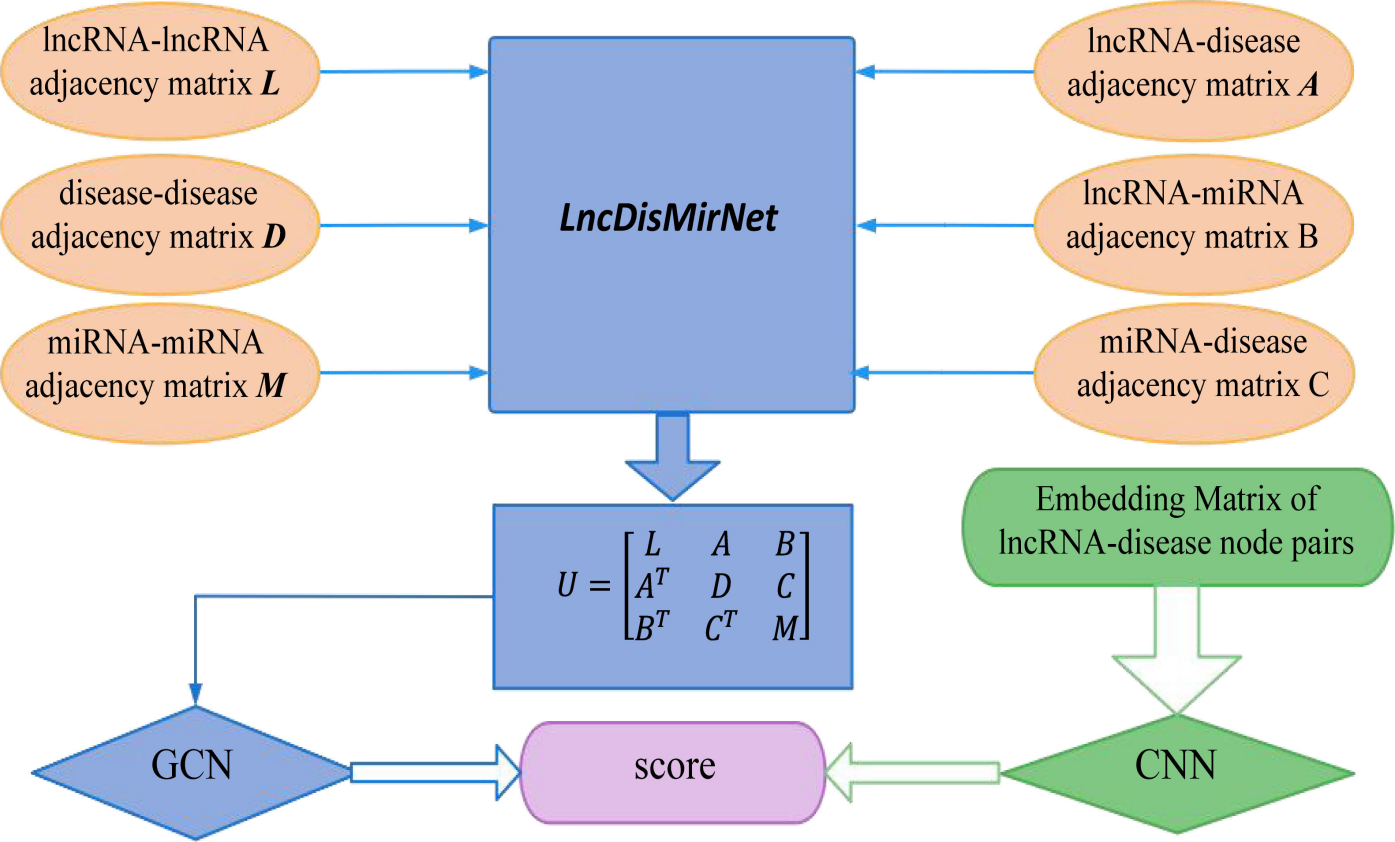
From the workflow, GCNLDA based on a graph convolutional network and a convolutional neural network was developed to learn network and local representations of the lncRNA-disease pairs.

Xuan et al. came up with a new method based on graph convolutional networks and convolutional neural networks, called GCNLDA, which is proposed for predicting lncRNA-disease associations (Xuan et al., 2019). **Figure 7** shows that GCNLDA proposes a new framework for learning network and local representation of lncRNA-disease pairs. The embedding matrix of lncRNA-disease nodes is constructed based on the biological premises of the analyzed lncRNAs, diseases, and miRNAs. Firstly, a lncRNA-miRNA-disease heterogeneous network named LncDisMirNet is constructed. the similarity of two lncRNA nodes is calculated using the method of Chen et al. When the similarity was > 0, an edge is used to connect the two lncRNA nodes, and the weight of the edge is set as the similarity value. The final calculated LncNet is denoted by *L*=[*L*_*i*,*j*_]∈*R* ^*Nl*×*Nl*^ , where *L_ij_* is the similarity between *l_i_* and *l_j_* and *N_l_* is the number of lncRNAs. The matrix *M*=[*Mi*,*j*]∈*R* ^*N*_*m*_×*N*_*m*_^ was used to represent the MirNet with *N_m_* miRNA nodes. The matrix *D*=[*Dij*]∈^*N*_*d*_×*N*_*d*_^ is a representation of DisNet network, and *N_d_* is the number of diseases. The linkage matrix *A*=[*Aij*]∈*R* ^*N*_*l*_×*N*_*d*_^ between LncNet and DisNet nodes was established using known LncRNA-disease correlation data, while the linkage matrix *B*=[*Bij*]∈*R* ^*N*_*l*_×*N*_*m*_^ between LncNet and MirNet and the linkage matrix *C*=[*Cij*]∈*R* ^*N*_*d*_×*N*_*m*_^ Between DisNet and MirNet were established based on the data of LncRNA-miRNA interactions and miRNA-disease associations. LncNet, DisNet and MirNet are combined to form the heterogeneous network of LncDisMirNet. LncDisMirNet consists of matrix

*U*=[*Uij*]∈*R* ^*N*×*N*^ :


(2) $$U=\left[\begin{array}{ccc} L & A & B\\ A^{T} & D & C\\ B^{T} & C^{T} & M \end{array}\right]$$

where *N*=*Nl+Nd+Nm*, and *A^T^*, *B^T^*, *C^T^* are transpose matrices of *A*, *B* and *C*, respectively. Besides, *u_i_* is the topological feature vector of the *i^th^* node in LncMirDisNet. Then an attention mechanism is established to extract the important features of LncRNA and disease nodes that can improve the association prediction. Then, the heterogeneous network *U* is used as an input to the graphical convolution $\tilde{U}$ The network *Z_i_* of lncRNA nodes and the network *Z_j_* of disease nodes obtained by combining the graph convolutional neural network. Finally, the prediction scores of the association between lncRNA and disease were obtained. Construction of LncRNA-disease node pair embedding matrix *P_i,j_*. The local representation of *l_i_-d_j_* is learned by using the embedding matrix *P_i,j_* of node pairs *l_i_*-*d_j_* as the input to the convolutional neural network for learning the marginal information of *Pi,j* in the convolution process. GCNLDA had the best performance for 405 diseases. The AUC of the ROC curve was 0.959. Case studies of gastric cancer, osteosarcoma and lung cancer confirm that GCNLDA effectively identifies potential lncRNA-disease associations.

**Figure 8:**
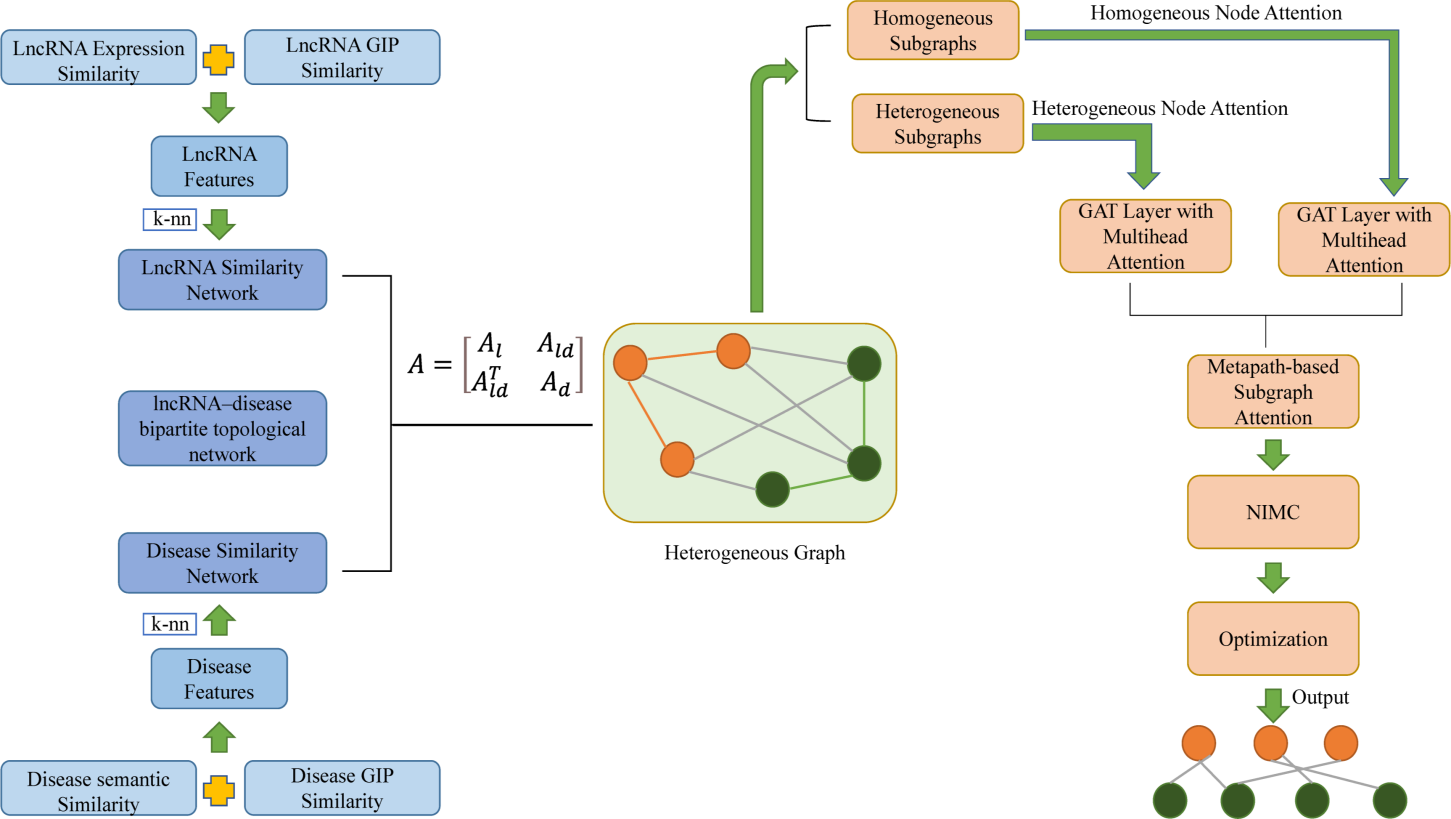
The workflow of predicting lncRNA-disease associations by heterogeneous graph attention network framework based on meta-paths (HGATLDA).

Zhao et al. proposed a novel heterogeneous graph attention network framework based on meta-paths (HGATLDA) to predict lncRNA-disease associations, inspired by a heterogeneous graph attention network (HGAT) (Zhao et al., 2022). As can be seen from **Figure 8**, lncRNA expression similarity and lncRNA Gaussian interaction profile kernel (GIP) (van Laarhoven et al., 2011) similarity were concatenated as lncRNA features. In the same way, disease semantic similarity and disease GIP similarity were concatenated as disease features. The lncRNA–disease bipartite topological network was slightly integrated with the lncRNA similarity network and disease similarity network which was generated by k-NN graph based on the lncRNA features and disease features, to generate heterogeneous graphs. HGATLDA is based on meta-paths, which is defined as a path in the form of $c_{1}\overset{R_{1}}{\to }c_{2}\overset{R_{2}}{\to }\ldots \overset{R_{l}}{\to }c_{l+1}$ , which denotes node type c_1_ and c_l+1_ is connected by a composite relation *R* = *R*_1_ ∘ *R*_2_ ∘ · · · ∘ *R*_*l*_ ,whererepresents the composition operator on relations. Specifically, the method first decomposes the heterogeneous graph into multiple subgraphs, homogeneous subgraphs and heterogeneous subgraphs were obtained based on whether the first and last node types of a meta-path are the same. GAT (Velickovic et al., 2017) is an effective tool for learning graph representations by assigning different weights to different neighbors. Then separately implement the GAT layer with multihead attention, different attention scoring ways were used for homogeneous subgraphs and heterogeneous subgraphs. Then the importance of each subgraph is learned and taking the semantic-specific node embeddings from metapath-based subgraphs as input. Subsequently, utilize NIMC (Li et al., 2020) to build a nonlinear neural rating model to reconstruct LDA matrix. Ultimate, cost-sensitive neural networks (Kukar and Kononenko, 1998) were incorporated to address the imbalance problem prevalent in LDA prediction, model learns parameters by minimizing a reshaped loss function and minimizes the loss function by ADAM optimizer. By comparing the HGATLDA method with some previous methods such as SIMCLDA, LAGCN by utilizing two kinds of 5-fold cross-validation (5-CV1 and 5-CV2). For 5-CV1, HGATLDA achieves the highest AUC value of 0.9424, which are 1.4% higher than the 2nd best of LAGCN. For 5-CV2, HGATLDA also achieves the highest AUC value of 0. 9262, which is 10.2% higher than the 2nd best of LAGCN. Case studies proved that HGATLDA has a good effect on LDA prediction. The advantages of HGATLDA include better model performance and the ability to extract more information from multiple biological data sources for LDA prediction.

Most prediction models do not adopt a single machine learning or deep learning method. Most models designed by predecessors adopt the integration of multiple machine learning methods or the integration of machine learning and deep learning. The combination of different classifiers in these integrated models and the different combination order of classifiers will affect the classification effect of the models.

## 6 Results

Here we comprehensively compared SIMCLDA, GCNLDA, LDICDL and other three models (**Table 1**). **Table 2** shows the top 5 lncRNA-disease associations that have been successfully experimentally confirmed in case studies of these models. The data sets used by the models being compared were downloaded from different databases by different models and processed in different ways. The data building process for the six models we compared is included in a supplementary materials document. Most of these models used lncRNA-disease associations supplemented with other associations as input, such as: lncRNA-miRNA associations, disease semantic similarity, disease-miRNA associations and others. Input data is then processed by GCN, KNN and other classifiers to predict the association between lncRNA and disease. Most of the training data of the models were obtained from the LncRNADisease database and other databases such as lnc2cancer database and HMDD database, then using LOOCV,5-CV and 10-CV to perform cross validation experiments on some widely used data sources or self-built databases, the predictive performance results of these models are provided. Most of the models obtain good prediction results: GCNLDA model obtains the AUC value of 0.9598, HGATLDA model obtains the AUC value of 0.9424. But SIMCLDA model obtain lower AUC values of 0.8526, which may be due to the fact that only the LncRNADisease dataset was used for training. All models are validated by case studies and the results are favorable. Furthermore, a completely independent test set should be constructed to objectively and comparatively evaluate the performance of different models.

**Table 1:** Machine learning models for predicting lncRNA and disease associations.

Model	Inputs	Classifier	Database	Case Studies	Evaluation methods	Performance(AUC)
SIMCLDA	lncRNA sequence similarity +disease–gene associations+ gene–GO associations+ the incomplete lncRNA–disease association matrix	inductive matrix completion	LncRNADisease	renal cancer, gastric cancer, prostate cancer	LOOCV	0.8526
iLncDA-LTR	lncRNA-disease associations+ disease semantic similarity+ lncRNA sequence similarity	Adaboost+ Xgboost+ k-NN+LTR	LncRNADisease/LncRNADisease v2.0	astrocytoma, breast cancer, hepatocellular carcinoma, prostate cancer, colorectal cancer	10-CV	0.9517
ENCFLDA	lncRNA-miRNA association+ miRNA-disease association	matrix decomposition+KNN	MNDRv2.0/HMDD/Starbase v2.0	breast cancer, lung cancer	LOOCV	0.9148
LDICDL	lncRNA-disease associations+ lncRNA-gene function associations+ lncRNA-miRNA associations+ disease-miRNA associations+ disease-gene associations	stacked denoising autoencoder+ matrix factorization	lncRNA2target /GeneRIF /starBase v2.0/HMDD /DisGeNET	osteosarcoma	10-CV	0.9134
GCNLDA	lncRNA disease associations+ lncRNA-miRNA interactions+ miRNA-disease correlations	GCN+CNN	LncRNADisease /lnc2cancer /GeneRIF/HMDD /Starbase/Dincrna	stomach cancer, osteosarcoma, lung cancer	5-CV	0.9598
HGATLDA	lncRNA-disease associations	GCN	LncRNADisease v2.0/Lnc2Cancer 3.0	breast cancer, hepatocellular carcinoma	5-CV1/5-CV2	0.9424/0.9262

**Table 2:** The top 5 lncRNA-disease associations that have been successfully experimentally confirmed in case studies of various machine learning models.

Model	lncRNA	Disease
SIMCLDA	H19, MALAT1, GAS5, MEG3, XIST	renal cancer
	MALAT1, DRAIC, PCAT29, GAS5, DISC2	gastric cancer
	HOTAIR, XIST, UCA1, NEAT1, SRA1	prostate cancer
iLncDA-LTR	NR2F1-AS1	astrocytoma, breast cancer, hepatocellular carcinoma, prostate cancer, colorectal cancer
	DLEU2	breast cancer, hepatocellular carcinoma, astrocytoma, colorectal cancer, prostate cancer
ENCFLDA	XIST, MALAT1, KCNQ1OT1, OIP5-AS1, NEAT1	lung cancer
	OIP5-AS1, SNHG16, SCAMP1, FGD5-AS1, LINC00657	breast cancer
LDICDL	H19, PVT1, GAS5, NEAT1, KCNQ1OT1	osteosarcoma
GCNLDA	MALAT1, NEAT1, MIR17HG, HOTTIP, TUG1	stomach cancer
	H19, GAS5, PVT1, NEAT1, EWSAT1	osteosarcoma
	KCNQ1OT1, HOTTIP, SPRY4-IT1, TP73-AS1, MIAT	lung cancer
HGATLDA	H19, MALAT1, MEG3, NEAT1, CDKN2B-AS1	breast cancer
	H19, MALAT1, NEAT1, CDKN2B-AS1, TUG1	hepatocellular carcinoma

## 7 Discussion and conclusion

Studies have shown that lncRNAs are involved in many important biological processes such as epigenetic regulation, cell differentiation, cell cycle regulation and immune response. In addition, mutations and dysregulation of lncRNAs are associated with many complex human diseases. Therefore, identifying lncRNA-disease associations (LDAs) has important biomedical implications, not only helping biologists to understand disease mechanisms, but also providing new ideas and solutions for diagnosis, treatment and prevention of diseases. Since traditional biological experimental and clinical methods are time-consuming and costly for identifying LDAs, scholars have shifted their research direction to efficient machine learning methods. Machine learning can accurately discover unknown LDAs and help guide future biomedical research. However, current models still have some limitations. In particular, the known data on lncRNA-disease associations are limited and no standard negative sample data are available, which leads to the construction of computational models that can only train known small-scale datasets. In addition, some machine learning algorithms are black-box learning algorithms that do not explain well the set of operations performed in the prediction process from a biological perspective. To overcome these drawbacks, we propose the following considerations for the construction of future prediction models for LDAs. First, a comprehensive database of LDAs needs to be constructed and a more suitable method for negative sample data construction needs to be found. A large amount of lncRNA-disease associations data and more reliable negative sample data will help to improve the accuracy of the prediction model. Then, more effective input features can be found to extract advanced features from the raw data, and the sequence, structure and physicochemical information of lncRNAs, etc. and more different heterogeneous network features can be tried to be applied as input features in lncRNA-disease associations prediction. In addition, most of the above prediction methods use the area under the ROC curve (AUC) and the area under the PR curve (AUPR) to evaluate the model performance, but these evaluation metrics may not fully reflect the merits of the model performance. Therefore, using more evaluation metrics can evaluate the prediction performance of the model more comprehensively, such as KS value, GINI coefficient, etc. Finally, for some machine learning models, the integration and feature selection of multiple biological data, the selection of optimal parameters and the combination of classifiers will also adjust the performance of the model. The above discussion will help us establish the lncRNA-disease association prediction model and provide ideas for us to design prediction models in other directions.

In this review, we briefly summarize and outline some of the popularly studied human diseases, association and similarity features of LDAs prediction, and performance evaluation methods of the models in LDAs prediction models. In addition, we comprehensively review some computational models based on machine learning methods that have been successfully applied to predict lncRNA-disease associations. We elaborate on the computational procedures of some methods and briefly explain the advantages and disadvantages of these methods. Finally, we discuss the potential limitations of machine learning-based methods for predicting LDAs and provide some other possible directions for designing reasonable prediction models. Although there have been several reviews on related topics, our review summarizes important diseases in LDAs prediction models, generalizes various similarity feature calculation methods, and introduces some new representative machine learning-based prediction methods. The content of our review complements the previously published review to some extent. Although this review is limited, it does not summarize all the studied diseases. It only introduces some machine learning-based models in LDAs prediction model and does not introduce models other than models that are not based on machine learning. We still expect that our review will contribute to a better understanding of lncRNA’s association with disease and further development of better-performing predictive models.

At present, the research on lncRNA-disease association prediction has attracted more and more attention, and the prediction methods based on machine learning are also increasing. However, there are still some challenges in lncRNA and disease association prediction. It mainly includes the following points:

(1) dataset. At present, there are limited data on the association of lncRNAs with disease. Most models use association matrix/network as input, and less known association data will limit the performance of the model.

(2) The construction of negative samples. It is difficult to know which lncRNA-disease pairs are not associated. The model based on machine learning needs to build negative samples, and can only build negative samples according to unknown associations, which will reduce the accuracy of the model.

(3) Input features. The input features of current prediction models are basically similarity features, and new similarity feature algorithms which are helpful to improve the prediction performance need to be developed. Designing new suitable feature inputs is a difficult problem to be solved.

(4) Adjustment of model parameters. The performance of the prediction model is closely related to the parameters of the model classifier, and the parameters are set differently for different data sets to obtain the optimal performance. At present, many models use manual parameter adjustment, so it is very important to find the method to obtain the optimal parameter.

Based on the above, in future studies on lncRNA and disease association prediction, we can start with data, association network and new lncRNA and disease association studies. We can mine more unknown disease-related lncRNAs by collecting more known relational data. At present, most prediction models need to build negative samples, and the negative samples built based on unknown interactions will inevitably reduce the prediction performance of the model. Therefore, it is a future development direction to design better unsupervised or semi-supervised training models that do not depend on negative samples. For the construction of semi-supervised models, we can refer to Chen et al. ‘s MiRNA-disease association prediction model (Chen et al., 2021; Wang et al., 2022b). The current input features are almost all the correlation matrix/network and similarity features of lncRNA and disease. We can get ideas from the prediction model of ncRNA-protein interaction (Wang et al., 2021), and try to combine the sequence and structural features of lncRNA, such as the one-hot coding features of lncRNA sequence, k-mer coding features and improved k-mer coding features are input to the prediction model together with the associated features. In conclusion, we hope to use more biological information and new machine learning models to develop more effective methods to predict lncRNA-disease associations in the future.

## Author contributions

XL, LZ, and ZD collected literature and wrote this review under the guidance of JT. JT, XL, LZ, and ZD were involved in revising it critically. All authors contributed to the article and approved the submitted version.

## Funding

This work was supported by the Beijing Natural Science Foundation (No. 2202002) and the National Natural Science Foundation of China (21173014).

## Conflict of interest

The authors declare that the research was conducted in the absence of any commercial or financial relationships that could be construed as a potential conflict of interest.

## Publisher’s note

All claims expressed in this article are solely those of the authors and do not necessarily represent those of their affiliated organizations, or those of the publisher, the editors and the reviewers. Any product that may be evaluated in this article, or claim that may be made by its manufacturer, is not guaranteed or endorsed by the publisher.
